# The Emergency Medical System (EMS) response to Iraqi pilgrims’ bus crash in Iran: a case report

**DOI:** 10.1186/s12873-019-0253-2

**Published:** 2019-07-16

**Authors:** Meysam Safi Keykaleh, Sanaz Sohrabizadeh

**Affiliations:** 1 Department of of Health in Disasters and Emergencies, School of Public Health and Safety, Shahid Beheshti University of Medical Sciences, Tehran, Iran; 2 Safety Promotion and Injury Prevention Research Center, School of Public Health and Safety, Shahid Beheshti University of Medical Sciences, Tehran, Iran

**Keywords:** Pre-hospital emergency, Mass casualty, Road traffic, Injuries, Iran

## Abstract

**Background:**

In Iran, Road Traffic Injuries (RTIs) with mass casualties occur repeatedly. Since Road Traffic Accidents (RTAs) occur far from health facilities, EMSs play an important role in reducing the disability and mortality resulting from RTIs. Thus, the study aimed to report Iraqi pilgrims’ bus which rolled over in the Malayer town.

**Case presentation:**

A mass casualty event occurred on 7 September 2017 when a bus full of Iraqi pilgrims rolled over on a road 4-km outside of Malayer, Iran. A large team of responders were dispatched including 5 ambulances with 10 EMTs along with 6 police officers serving in the area. The accident resulted in 35 injured patients (21 female and 14 male) as well as 11 deaths ranging in age from 2 to 65 years. Twenty-one of the injured were transported to the hospital and 14 patients refused transport and 12 patients sustained multiple trauma. The case has been described four phases of dispatch, on-scene, hospital and post-mission. Frequent calls made by laypeople were considered as the main challenge of dispatch phase. The response on scene was hampered by large numbers of lay bystanders. The over-crowding around the emergency units disrupted the medical care procedures in hospital phase.

**Conclusion:**

This case highlights over-crowding and laypeople interference at the scene disrupts the relief and rescue. To solve these challenges, the public education and police monitoring and control is recommended. Establishing a unified command post at the scene can facilitate effective coordination among relief and rescue organizations.

## Background

WHO report, Road Traffic Injuries (RTIs) are one of the most important health problems throughout the world [1] and the second cause of death in Iran [2–5]. Since deaths caused by RTIs can occur far from health facilities [6], Emergency Medical Systems (EMSs) play an important role in reducing the disability and mortality resulting from RTIs [7–10]. Prehospital teams face many challenges when RTIs result in mass casualties [5, 6]. The large number of RTIs in Iran as well as the EMS primary role to provide medical assistance make, reporting on the unusual conditions in which emergency medical services are provided in RTIs in Iran is important for improving pre-hospital services [11]. This manuscript presents the case of a bus rollover in Malayer, Iran which resulted in a large number of casualties. It uses this incident to describe challenges faced in the prehospital response and to highlight areas for continued improvement of prehospital services.

## Case presentation

On 7 September 2017 a bus full of Iraqi pilgrims rolled over on a highway 4-km outside the town of Malayer and 9 km away from the nearest hospital. Five ambulances with 10 Emergency Medical Technicians (EMTs), 2 crane trucks with 5 technicians along with police officers serving in the area (Table 1). The accident resulted in 35 injured patients (21 female and 14 male) as well as 11 deaths ranging in age from 2 to 65 years. The hospital while 14 others refused transport. The majority of patients sustained multiple injuries (Table 2).

**Table 1:** The EMS Response based on Time Intervals and distances

Ambulance ID number	Distance between EMS station and scene (mile)	Distance between scene and hospital (mile)	Ambulance departure time	Arrival time at scene	Leave scene time	Arrival time at hospital	Hospital ID number	Delivery time	Number of transported casualties (N)
1	5	5.6	18.48	19:00	19:20	19:27	1	19:55	4
2	5	5	18:49	19:00	19:46	19:54	2	20:00	5
3	3.72	5	19:00	19:08	19:20	19:27	3	19:32	3
	–	5.6	19:33	19:41	19:59	20:07	1	21:40	4
4	13:67	5.6	18:50	19:03	19:48	20:00	1	20:20	4
5	5	–	19:29	19:38	21:10	–	–	21:10	–

Hospital 1: Trauma center

Hospital 2, 3: General

**Table 2:** The frequency of Trauma Type among Iraqi Pilgrims’ Bus Crash

Category	sub-category	Frequency	%
Multiple trauma	Chest and right and left humerus trauma	2	5.71
	Head and abdominal trauma	2	5.71
	Head trauma and ulna and radius fracture	1	2.85
	Head and spinal cord injury	5	14.28
	Head trauma and fingers, wrist injury	1	2.58
	Head and spinal cord injury and tibia and fibula fracture	1	2.58
Single trauma	Chest trauma	1	2.85
	Limb trauma	7	16.31
Without chief complaint		15	42
Total		35	100

### Dispatch phase

Relief activities began with frequent calls made by laypeople. Two ambulances were dispatched immediately. The dispatch operator requested the callers to make a phone call to the police because he was answering four (phone) lines at the same time. The operator, however, could get through to the police center only after several minutes. Since the EMTs had to deal with many victims, there was an urgent need to recall the off personnel; however, the dispatch operator could not reach them as a result of lacking a recall plan and protocol. Some drivers did not hear the ambulance alarms and made it more challenging for the EMTs to arrive at the scene. Furthermore, the police officers, EMTs and firefighters were not all dispatched at the same time.

### On-scene phase

The response on scene was hampered by large numbers of lay bystanders who disrupted rescue and transport procedures and complicated the scene triage process by which patients were evaluated for salvageable injuries. Some of these bystanders were agitated and were aggressive toward the EMTs. Multiple instances of taking photos and videos from the scene were observed. Rescue was delayed because of the firefighters’ late arrival. Accordingly, some EMTs entered the rolled-over bus to attempt to rescue passengers trapped inside despite the ongoing risk from leakage of gasoline after the accident. The casualty triage was ineffective resulting in low acuity “green” tagged patients being transported higher acuity “red” patients.

Laypeople put corpses into ambulances. The lack of wireless telephone services was considered a communication challenge for all the relief and rescue providers on the scene. Insufficient coordination among the police officers, EMTs and firefighters was also observed at the scene.

### Hospital phase

While several of the medical staff (MDs and nurses) at the hospitals had a suitable collaboration with the EMTs and managed the injuries well in the emergency units, some others behaved disrespectfully toward the EMTs. The over-crowding around some of the outpatient casualties at the emergency units disrupted the medical care procedures.

### Post-mission phase

At the end of the EMTs’ mission, the police called the EMS dispatch center and reported that a 12-year-old child was found by a local rural resident. The boy was shocked and did not talk to anybody. The EMTs found that he was a pilgrim who had run away from the scene after the accident.

## Discussion and conclusion

Some people neglected the ambulance sirens, contributed to a heavy traffic near the scene and congested the accident site. Congestion can disrupt first aid services for victims [12] and negatively affect the arrival and response time at the scene [13–15].

The non-integrated management of the various involved organizations as well as the lack of a unified incident command post resulted in ineffective relief and rescue operations at the scene. Several other studies have also reported a lack of coordination among relief and rescue organizations [16–18].

The delayed arrival of the police officers and firefighters resulted in the inadequate security and safety of the EMTs and victims. The police are expected to be the first force ensuring accident scene security [19].

In this case, the EMS dispatched five ambulances with ten crews. Meanwhile, the case report of a bus crash in Switzerland (March 13, 2012) stated that eight rescue helicopters, 15 physicians, 100 paramedics and three psychologists were dispatched to the accident scene of 46 victims [20]. The lack of a recall plan for off personnel affects the sufficient supply of EMS workforce.

Although studies on the subject have shown that triage is a basic skill for EMTs [19, 21], in this case, the EMTs could not correctly triage the injured people at the scene. The bystanders’ interferences and the EMTs’ inadequate skills may have resulted in such a weak triage. It seems that first-aid rules and skills should be taught to communities.

Fear of the bus exploding, the provision of first-aid services for a considerable number of casualties, transporting several of the casualties by a single ambulance, the disrespectful behaviors of some physicians and nurses at the time of the victims’ delivery to the hospitals as well as violence against the EMTs were observed as some of the factors that affected the EMTs’ mental health. Psychological disorders among the EMS personnel were also reported as a result of having to provide medical care to multiple victims [22–25]. The over-commitment of several EMTs to the extrication of trapped victims endangered their own life. Although this level of commitment among the EMTs can be considered a positive opportunity for EMS managers [26], several studies have reported that over-committed behaviors can threaten EMTs’ safety [26, 27]. Establishing supervision rules and training for over-committed EMTs who put themselves in danger to save victims are suggested. The following lessons can be learnt from this case report:

- Personnel recall plan needs to be mandatory and put in place and practiced by all pre-hospital emergency centers.
- To solve the challenges of over-crowding and interferences of lay bystanders at the scene, public training and police monitoring and control are recommended.
- To decrease the response time, it is recommended to dispatch several ambulances to the scene in the case of a bus crash rather than to dispatch one ambulance for rapid assessments.
- Establishing a unified command post at the scene is suggested to facilitate effective coordination among relief and rescue organizations.
- Hospitals should have a pre-emergency plan as well as specialized training and exercising for the management of mass casualty incidents and people labeled with a green tag.
- Preparing and comparing a list of passengers with the number of victims can be helpful for finding the lost ones in cases such as bus accidents.

## Data Availability

Not applicable.

## Abbreviations

EMS: Emergency Medical Services
EMTs: Emergency Medical Services
MD: Medical Doctor
RTIs: Road Traffic injuries
